# Novel Approach to Assess the Toxicity of PM_2.5_ on a Subway Platform Using a Mobile Air–Liquid Interface System and Submerged Exposure

**DOI:** 10.1021/acsestair.6c00018

**Published:** 2026-07-01

**Authors:** Micol Introna, Ana Teresa Juárez-Facio, Naga Veera Srikanth Vallabani, Minghui Tu, Michael Norman, Sanna Silvergren, Andrea Colombo, Valentina Liboni, Bozhena Tsyupa, Alessandro Mancini, Ulf Olofsson, Sarah Sulamith Steimer, Hanna Lovisa Karlsson, Karine Elihn

**Affiliations:** † Department of Environmental Science, 7675 Stockholm University, 106 91 Stockholm, Sweden; ‡ Institute of Environmental Medicine, Karolinska Institute, 171 77 Stockholm, Sweden; § Department of Machine Design, 7655 KTH Royal Institute of Technology, 100 44 Stockholm, Sweden; ∥ Environment and Health Administration, SLB-analys, 104 20 Stockholm, Sweden; ⊥ Department of Environmental Health Sciences, Istituto di Ricerche Farmacologiche ″Mario Negri″- IRCCS, 20156 Milano, Italy; # GCF Research & Development, 476077 Brembo NV, 24040 Stezzano, BG, Italy

**Keywords:** subway, railway, particulate matter, inflammation, air−liquid interface, submerged exposure

## Abstract

Subway stations are enclosed environments that can have elevated concentrations of PM_2.5_ (particulate matter with an aerodynamic diameter of <2.5 μm). These particles are primarily generated by mechanical wear from trains and are therefore rich in transition metals and suspected to affect health upon inhalation. To simulate real-world inhalation scenarios, this study aimed to assess the toxicity of airborne subway PM_2.5_ using a mobile air–liquid interface (ALI) exposure system directly on the platform, using monoculture (A549) and coculture (A549 + dTHP-1) cell models, alongside laboratory submerged exposures. In ALI exposures, a dose of 0.12 ± 0.07 μg/cm^2^ could not induce any change in cell viability or inflammatory markers after 24 h of incubation. In submerged experiments, a higher dose was applied than in ALI, inducing a decrease in cell viability in both A549 and dTHP-1 cells. A549 cells exhibited increased IL-6 and TNF-α release at the highest dose (100 μg/mL). In dTHP-1 cells, IL-8 release was significantly elevated even at the lowest dose (10 μg/mL), and all four cytokines (IL-8, IL-6, IL-1β, and TNF-α) were significantly increased at 100 μg/mL. These findings highlight the critical role of exposure conditions in toxicological studies and support the use of both *in situ* and laboratory-based approaches. Moreover, longer *in situ* exposures may be necessary to achieve doses comparable to those in a laboratory setup for comparison.

## Graphical abstract

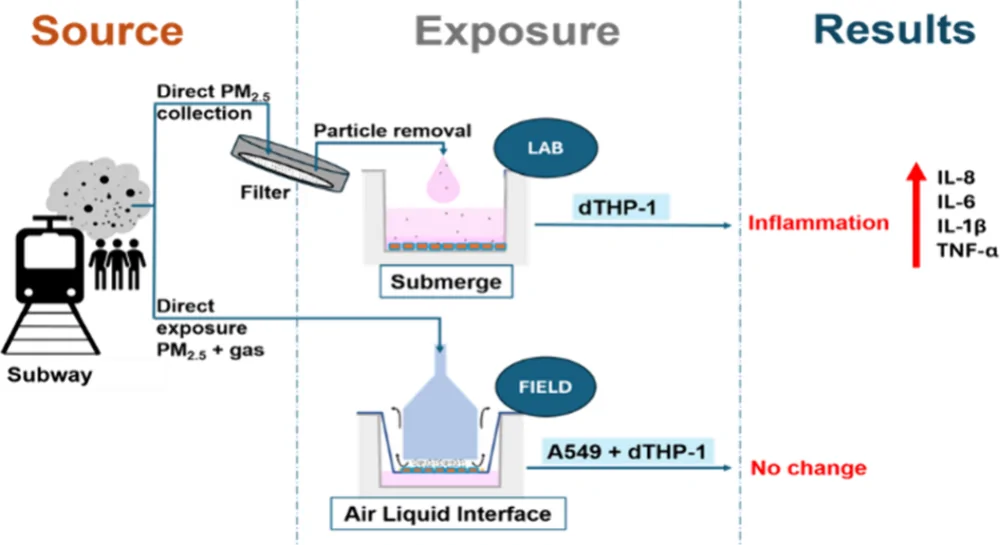

## 1 Introduction

Subway systems offer rapid and safe transport, improving a city’s efficiency by developing underground and thereby preserving surface space for urban planning. In metropolises such as Shanghai, New York, Stockholm, and Toronto, it is estimated that 7.7, 5.4, 1.3, and 1.1 million people, respectively, use the subway every day.^1–4^ According to recent predictions based on the United States Environmental Protection Agency (EPA) risk assessment model, subway air pollution is associated with both carcinogenic and noncarcinogenic risks to human health.^5^

When comparing urban environments such as streets and subways, PM_2.5_ (particulate matter with an aerodynamic diameter of <2.5 μm) differs in both composition and concentration due to different emission sources and dispersion conditions. Street-level PM_2.5_ is primarily emitted from exhaust emissions,^6^ whereas subway PM_2.5_ is dominated by nonexhaust sources.^7^ In addition, fuel combustion tends to dissipate more rapidly in the open outdoor environment at street level,^8^ while subway PM_2.5_ is confined within tunnels and platforms due to the limited ventilation.^9^ As a consequence, in Stockholm underground subway stations, the concentration of PM_2.5_ was measured to be higher than on the street level.^10,11^

In the subway, the sources of PM_2.5_ are primarily determined by the friction of the train wheels, brakes, and rails, as well as the sliding of electric cables.^7^ Consequently, subway PM_2.5_ often contains large mass fractions of metals: Fe, Mn, Cu, Pb, and other elements (e.g., Si, S, and Br).^10–12^ Based on this fact, a brief stay in the metro of 70 min contributes to about 90% of the daily exposure to metals.^3^ Depending on their oxidation state, inhaled metals can dissolve into metal ions that facilitate the generation of reactive oxygen species (ROS), e.g., free hydroxyl radicals. Specifically, Fe­(II) induces the production of ROS through the Haber–Weiss and Fenton reactions; similarly, Cu­(I) can produce free radicals by being oxidized to Cu­(II).^13,14^

Although the number of cities with subways is expected to grow, only a few *in vitro* studies have examined the effects of subway PM_2.5_ on the respiratory system. Those toxicological investigations have mainly been conducted by sampling PM_2.5_ and PM_10_ on filters in the subway and exposing lung cells to particle suspensions under submerged conditions.^15–20^ In Stockholm, Karlsson et al.^24^ compared the *in vitro* toxicity between subway and street-level particles. In cells treated with subway particles, the genotoxic response was eight times higher compared to those exposed to road particles. These particles had a high content of iron oxides, such as magnetite (Fe_3_O_4_) and hematite (Fe_2_O_3_); nevertheless, the enhanced toxic effect of the subway particles was suggested to be determined by factors such as coordination of Fe/Fe-ions on the particle surface.^21,22^ This leads to the induction of DNA damage, genotoxicity, and inflammation through oxidative reactions.^20,23–25^ The increased oxidative stress induced by PM from the subway on human cells was further corroborated by a study conducted in the Barcelona subway.^19^ In this study, Cu and Sb were identified as being primarily responsible for the increased oxidative potential (OP), whereas Fe, although predominant, did not appear to correlate with the increased oxidative stress. Finally, singular sources of subway-related nanoparticles composed of approximately 90 wt % iron exhibited limited capacity for ROS generation; however, those with higher silicon content (originating from the third rail) were associated with an increase in IL-8 gene expression.^26^ Discrepancies among studies on which metal primarily drives toxicity underscore the importance of preserving the original composition and physical properties of PM in toxicity assessments. *In vitro* exposure models, such as the air–liquid interface (ALI), are highly relevant, as they directly expose cells to airborne particles, preserving the particles' airborne state and allowing for the investigation of both the gas and particle fractions. In a subway study by Loxham et al.,^18^ pseudo-ALI exposures were performed using a very limited suspension volume of collected particles. To date, no ALI study has been conducted directly in the subway.

This study presents a novel approach to investigating the toxicity of subway PM_2.5_. To our knowledge, it is the first study to perform *in situ* experiments directly on a subway platform, enabling real-time exposure of lung cells to airborne particles. To achieve this, we placed the electrostatic air–liquid interface exposure (ELLIE) system,^27^ designed for testing the toxicity of air pollutants at outdoor locations, on a subway platform in Stockholm. This mobile ALI system directly deposits airborne PM_2.5_ from the source onto human lung cells and was previously used in a Stockholm road tunnel to perform direct cellular exposures to traffic-related PM_2.5_.^28^ The results were compared with submerged lung cell experiments, which used particles collected on filters during the same period as that of the ALI direct exposures. Detailed chemical analyses were also conducted to enhance our understanding of the current organic and inorganic composition of subway particles.

## 2 Materials and Methods

### 2.1 Exposure Location and Setup

A mobile ALI system, called ELLIE,^27^ was located on the subway platform at the Tekniska högskolan station in Stockholm.

The experimental setup used in this study was previously applied in a road tunnel in Stockholm to assess the toxicity of traffic-related PM_2.5._ ^28^ Briefly, the ALI system was placed on the platform and sampled at a flow rate of 1.5 L/min during the 2-h exposure (Figure 1). To achieve a high particle dose on the cells, a concentrator was placed upstream of the ALI system, delivering 1.5 L/min to the ALI, increasing the aerosol particle concentration approximately 5-fold.^28^ After the concentrator, a PM_2.5_ cyclone was used to select the particle size to be delivered to the cells. The selected PM_2.5_ was dried using a drier (MD-110-12S-4, Perma Pure, Lakewood, USA). The particle size distribution entering the exposure chamber was determined using an optical particle counter (OPC; aerosol spectrometer 1.109, Grimm Technologies, USA) that sampled 0.3 L/min from the main flow. The PM_2.5_ main flow was charged, humidified (RH > 90%, MH-110-12S-4, Perma Pure, Lakewood, USA), and delivered to seven separate wells containing cells cultured at the ALI. Three wells were used to assess the toxic effects of airborne PM_2.5_, while three additional wells served as internal controls, receiving only the gas phase (the aerosol was prefiltered using Headline filters, grade DIF-LN40). The final well was used to determine the dose. The flow in the wells (214 mL/min/well) was controlled via critical orifices positioned after each exposure chamber. An alternating high voltage (±1 kV) was used underneath the exposure chamber to increase the deposition of particles.

**Figure 1:**
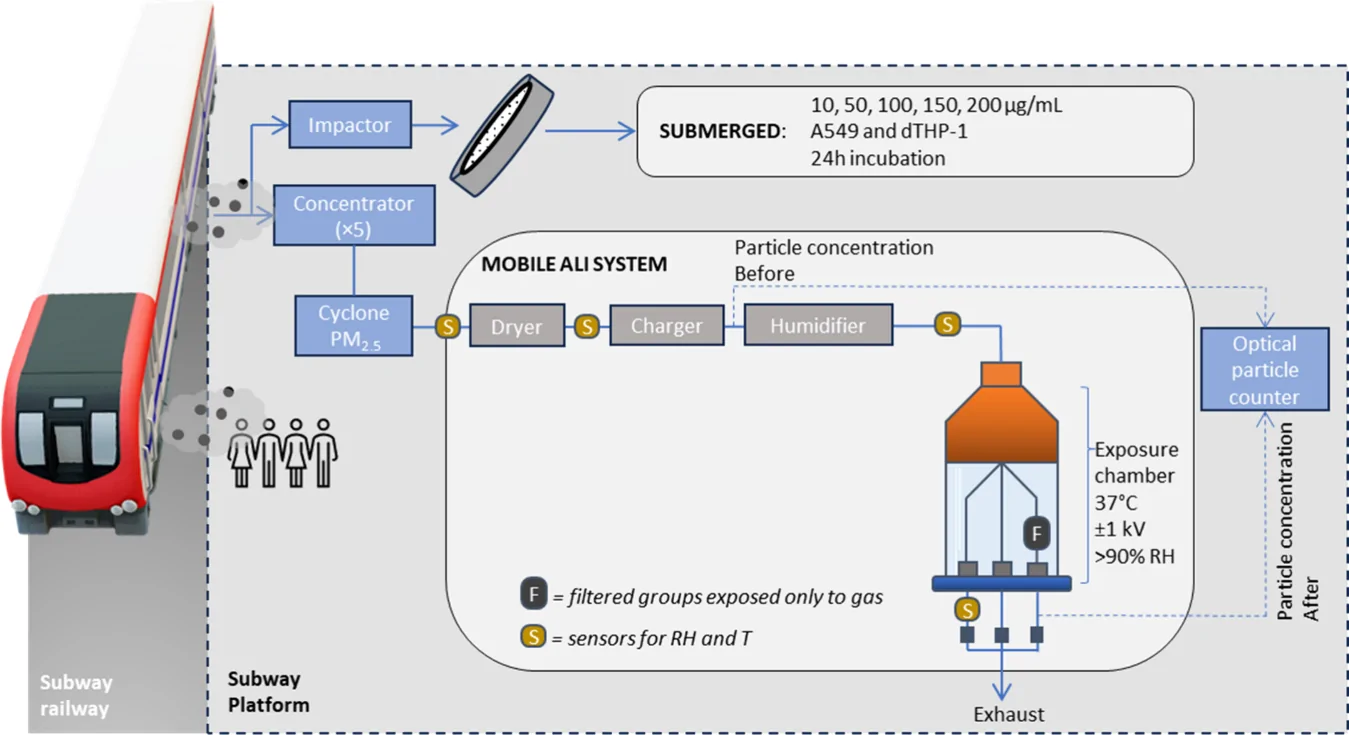
Schematic overview of the experimental setup and location in the subway.

The PM_2.5_ mass concentration was measured by using an aerosol spectrometer (Fidas 200S) during the experiments.^29^ Ambient total particle number concentration (PNC) was measured in parallel with the ALI exposures using a condensation particle counter (CPC; TSI model 3775) with a D50 of 4 nm. The instrument was housed in a temperature-controlled container, and data were recorded as 15 min averaged values. These values were analyzed to estimate the mean PNC (no./cm^3^) in the subway during each 2-h ALI experiment and to monitor the daily variation in PNC from March to June 2023.

### 2.2 Subway PM_2.5_ Filter Collection and Use in Submerged Experiments

PM_2.5_ was collected on PTFE filters (0.3 μm pore size) using a Dekati DGI impactor, following the ISO 23210:2009 standard.^30^ The collected particles were then used to test toxicity in a submerged model and for chemical analyses. The sampling method was previously described by Introna et al.^28^ Briefly, the impactor used a flow rate of 70 L/min and was set up without the foil cutoff stages to allow collection of PM_2.5_ on the PTFE end filter. The filters were stored in a climate-controlled room for 24 h before weighing.

### 2.3 Cell Culture for ALI and Submerged Conditions

For ALI exposure, A549 cells (ATCC CCL-185) were cultured and prepared following the same protocol as described by Introna et al.^28^ Briefly, cells were maintained in DMEM supplemented with 10% FBS, 1% sodium pyruvate, and 1% penicillin/streptomycin at 37 °C and 5% CO_2_. Cells were seeded onto semipermeable inserts (6.0 × 10^5^ cells/cm^2^ in 1.5 mL, Falcon Transwell surface 4.2 cm^2^) and, after reaching 80–90% confluence, placed under ALI conditions by removing the apical medium and replacing the basal medium with DMEM supplemented with 25 μM HEPES (4-(2-hydroxyethyl)-1-piperazine ethanesulfonic acid) prior to transport and exposure.

Cocultures of A549 and THP-1 cells (ECACC; Sigma–Aldrich, CB-88081201; leukemic monocyte) were also used in ALI. Both cell models were cultured separately in RPMI-1640 medium with 10% FBS, 2 mM l-glutamine, and 1% penicillin–streptomycin. THP-1 cells were cultured in RPMI-1640 medium with PMA (300 ng/mL) for 48 h to induce differentiation (hereafter called dTHP-1). A549 cells were seeded (6.0 × 10^5^ cells/cm^2^ in 1.5 mL) at the apical side of the cell inserts and placed in a 6-well plate with 2 mL of basal culture medium for 24 h. The day after, A549 cells were put under ALI conditions, and a new medium (1.5 mL) was added to the basal side with 25 μM HEPES. dTHP-1 was added to each insert at a ratio of 1:10 on top of A549 cells in a total volume of 1 mL. After 3 h, the apical medium was removed and cells were incubated in ALI for 20 h for adaptation.

Monocultures of A549 and dTHP-1 cells were used in the submerged exposure experiments as previously described.^28^ They were seeded in 96-well plates (Sarstedt AB, 1.0 × 10^4^ A549 cells/well, 24 h incubation; 55 × 10^4^ THP-1 cells/well with PMA 50 ng/mL, 48 h differentiation time). After incubation, cells were exposed (24 h) to suspensions (100 μL) of different particle concentrations.

### 2.4 Exposure to Subway PM_2.5_

#### 2.4.1 Exposure at ALI

Cells maintained under ALI conditions were transported from the laboratory to the subway platform in a temperature-controlled container. The transport to the exposure site was completed in approximately 15 min, a duration previously validated as having no adverse effects on cell viability.^27^

Cells were exposed for 2 h to subway PM_2.5_ aerosol (Exposed group) or to subway gas phase (Filtered group) using the same ALI exposure protocol described in Introna et al.^28^ Prior to exposure, 3.3 mL of fresh basal medium (DMEM for A549 monoculture or RPMI-1640 for coculture), supplemented with 25 μM HEPES, was added to each exposure chamber. Incubator controls were maintained at 37 °C and 5% CO_2_. After exposure, inserts were transferred to fresh basal medium and incubated for 24 h. Basal media were then collected, centrifuged (1500 rpm, 5 min), and stored at −80 °C for cytokine analysis, and cell viability was assessed using the Alamar Blue assay. In total, nine experiments were performed (four monoculture and five coculture).

#### 2.4.2 Exposure at Submerged Conditions

PM_2.5_ suspensions were made by removing particles via vortexing (3–5 min) of the PTFE filters in sterile Milli-Q water. Particle removal was determined gravimetrically after filter drying. FBS was added to the particle suspension to reach a final concentration of 5%. Negative control filters were processed identically. Stock suspensions were sonicated for 20 min, and working concentrations of 10, 50, 100, 150, and 200 μg/mL (which equals a nominal dose of 3–62 μg/cm^2^ at 100% deposition efficiency) were prepared immediately prior to cell exposure.

### 2.5 Dose Estimation in ALI

The number concentration of airborne particles was recorded using an OPC (aerosol spectrometer 1.109, Grimm Technologies, USA, aerosol flow rate of 1.2 L/min) as previously described.^28^ Briefly, the deposited particle number dose onto cell cultures was estimated from the difference in PNCs measured upstream and downstream of the ALI system, considering particles with sizes ranging from 0.25 to 2.5 μm. The particle count was then normalized by the cell insert surface area to determine the number of particles per cm^2^. The deposited mass dose on cell cultures was calculated by combining the total particle volume (OPC data) with the effective density of subway PM_2.5_ (13.4 g/cm^3^), measured using a Dekati DGI impactor (ISO23210:2009), based on the approach of Tu and Olofsson.^29^

### 2.6 Toxicological End Points

#### 2.6.1 Cell Viability

After incubation, cell viability was assessed using Alamar Blue in ALI and submerged exposure experiments as previously described.^28^

For ALI conditions, cells were incubated with the 10% Alamar Blue solution for 3 h. Thereafter, four 100 μL aliquots of apical solution from each insert were dispensed into a 96-well plate, and absorbance was measured at 570 nm using a spectrophotometer (CLARIOstar). Triton (0.1%) served as a positive control. Cell viability for each condition was expressed as a percentage relative to cells maintained under standard incubator conditions (negative control).

In submerged experiments, the exposure suspension on top of dTHP-1 and A549 was replaced by 10% Alamar Blue and incubated for 2 h under standard conditions. Co NP (50 μg/mL) served as the positive control. The fluorescence intensity was determined with a Tecan Infinite F200 plate reader (Tecan, Magellan 7.2 Software, Grodig, Austria) at 540/590 nm excitation/emission.

#### 2.6.2 Cytokine Release

Cytokine release was assessed for both ALI and submerged exposures. After 24 h of incubation, culture supernatants were collected, centrifuged (1500 rpm, 5 min), and stored at −20 °C (ALI) or −80 °C (submerged). For ALI samples, IL-1β, IL-6, IL-8, and TNF-α were quantified by ELISA. For submerged samples, cytokines were measured using the V-PLEX Pro-inflammatory Panel II. Samples were diluted as required, depending on the cell model and cytokine levels. Lipopolysaccharide (LPS) was used as a positive control in both ALI and submerged exposure experiments.

### 2.7 Analysis of Inorganic Fraction

#### 2.7.1 SEM–EDS Analysis

The shape and elemental content of subway PM_2.5_ particles were characterized using scanning electron microscopy combined with energy-dispersive X-ray spectroscopy (SEM–EDS), following the same procedure as described in Introna et al.^28^ Briefly, particles were recovered from filter halves by sonication in isopropanol, dried, mounted on carbon tape-coated aluminum stubs, and analyzed using a Zeiss EVO MA10 scanning electron microscope equipped with an EDS detector. Five representative areas were examined for each sample.

#### 2.7.2 XRD Analysis

The crystalline phases of subway PM_2.5_ particles were characterized by X-ray diffraction (XRD), following the same analytical approach as described in Introna et al.^28^ Measurements were performed using a Rigaku SmartLab diffractometer with Cu Kα radiation in Bragg–Brentano geometry, scanning the 2θ range of 5°–90°. Identification of crystalline phases was conducted using the PDF4+ crystallographic database (ICDD).^31^

### 2.8 Analysis of Organic Compounds

Organic compounds of subway PM_2.5_ particles were determined using an analytical procedure based on established EPA methods, as detailed in Introna et al.^28^ Samples were spiked with isotopically labeled internal standards and extracted by pressurized liquid extraction, followed by purification using gel permeation chromatography and solid phase extraction. Compounds were analyzed by GC-Orbitrap MS, with separation achieved on dedicated capillary columns for PAHs/OPAHs/PCBs and PBDEs. Quantification was performed using compound-specific calibration curves, and identification was based on retention times, mass spectra, and isotopic ratios. Detailed information on the analytical procedure is presented in the Supporting Information, Section S1.

### 2.9 Statistical Analysis

Statistical analyses were performed using GraphPad Prism (v. 9.0.3), applying one-way ANOVA and unpaired *t* tests, with a significance threshold of *p* ≤ 0.05. Data are presented as mean ± standard deviation from at least three independent exposures.

## 3 Results

### 3.1 Particle Mass and Number Concentration Measured during Subway Exposure Periods

The particle mass concentration (μg/m^3^) and number concentration (#/cm^3^) were measured on the platform between March and April 2023. Results of the mean PM_2.5_ (μg/m^3^) and PNC (#/cm^3^) measured during each of the nine 2-h ALI exposures conducted on the platform are presented in Figure 2. The particle mass and number concentration were consistent, with only minor fluctuations observed between experimental days. The daily variation in PNC from March to June 2023 is presented in Supplementary Section S2, Figure S1.

**Figure 2:**
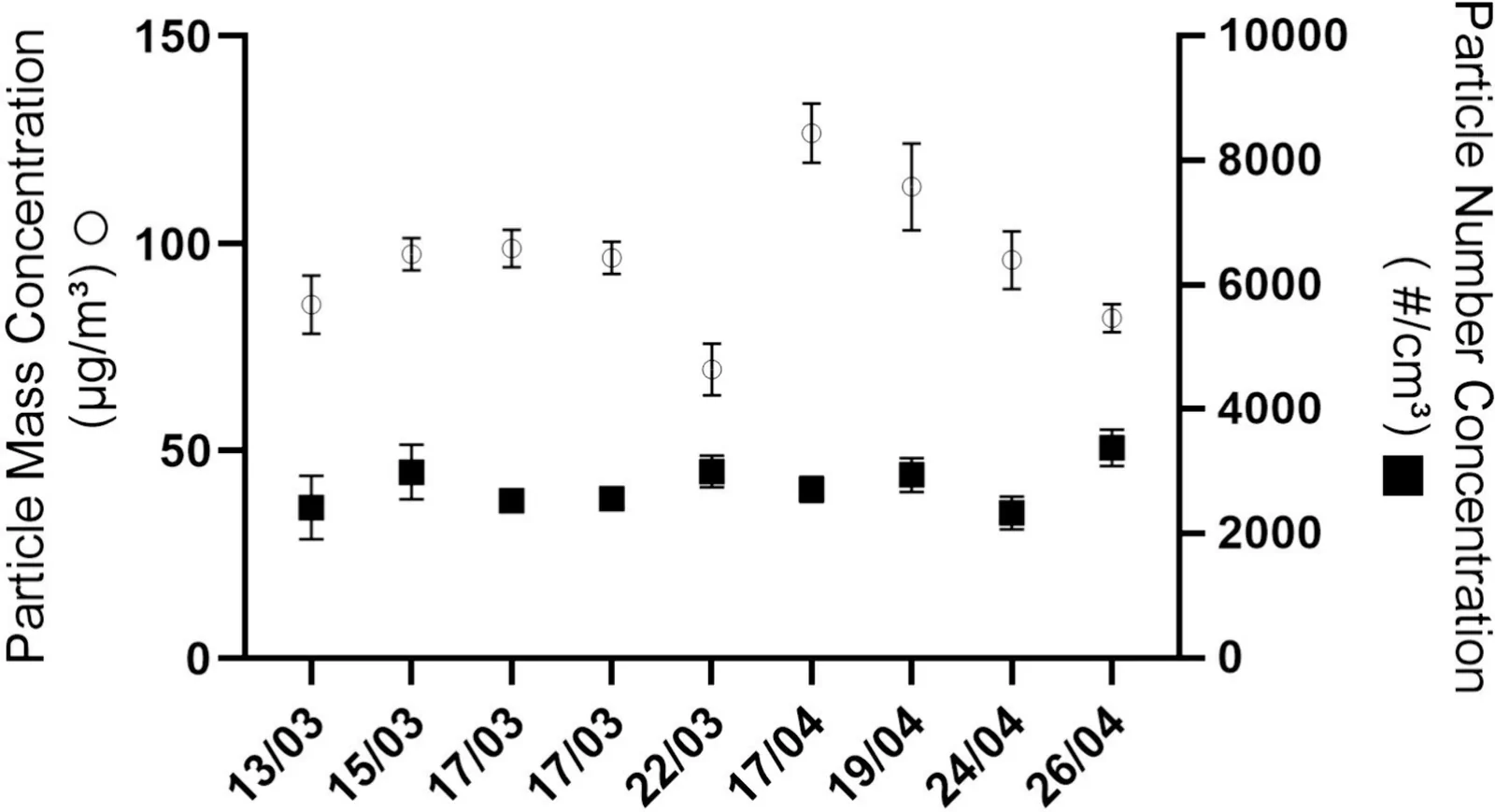
PM_2.5_ (μg/m^3^) and PNCs (#/cm^3^) for each ALI experiment. Values are presented as mean ± standard deviation for 2-h exposures. The *x*-axis shows the dates of the experiments.

### 3.2 Toxicological End Points

#### 3.2.1 Cell Viability in ALI and at Submerged Conditions

Cell viability following exposure to subway particulate matter in the ALI system was evaluated using the Alamar Blue assay in human lung A549 cells in monoculture, as well as in coculture with dTHP-1 macrophages. In both experimental cell models, no significant reduction in cell viability was seen compared to the incubator control (Figure 3: Control group), suggesting that the cells remained unaffected by the transport process and the exposure to particles (Figure 3: Exposed group) and gases (Figure 3: Filtered group).

**Figure 3:**
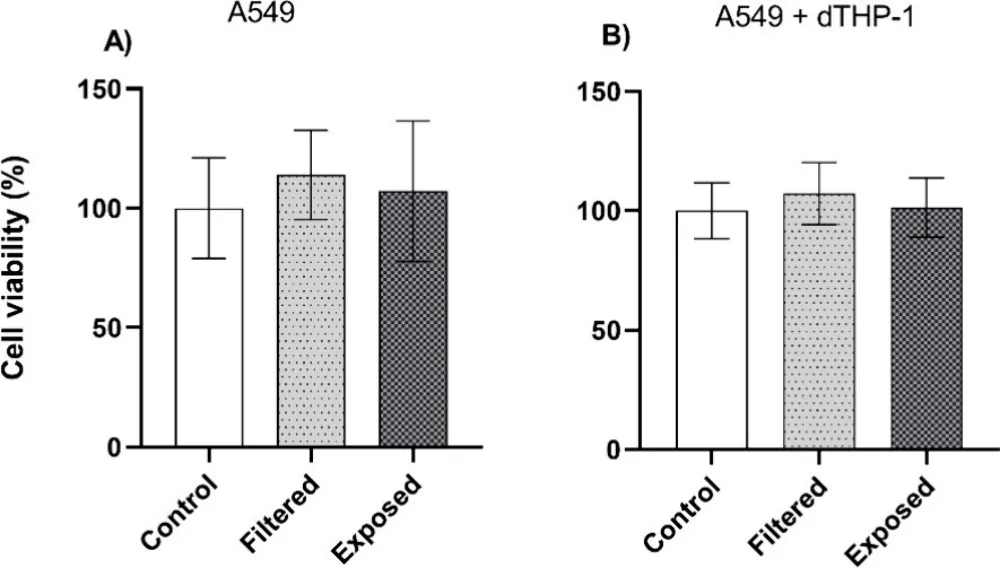
Cell viability of cells exposed to PM_2.5_ in ALI from a subway station in Stockholm. (A) A549 and (B) A549 and dTHP-1 coculture under ALI conditions versus control after being exposed to 0.12 ± 0.07 μg/cm^2^ for 2 h + incubated for 24 h. Results are presented as mean ± standard deviation of at least three independent exposures. Control = incubator control; Filtered = gas phase only; and Exposed = complete aerosol.

Submerged experiments were conducted by exposing A549 and dTHP-1 monocultures to subway PM_2.5_ collected on filters. These filters were sampled during the same period as the ALI exposures were performed, ensuring a comparable particulate mixture across both exposure models. In both A549 and dTHP-1, the highest doses (150–200 μg/mL) led to a statistically significant and dose-dependent reduction in cell viability. The cell viability was approximately 91 and 87% for A549 cells (Figure 4A) and 70 and 53% for dTHP-1 cells (Figure 4B) when using the 150 and 200 μg/mL exposure doses, respectively, compared to the control.

**Figure 4:**
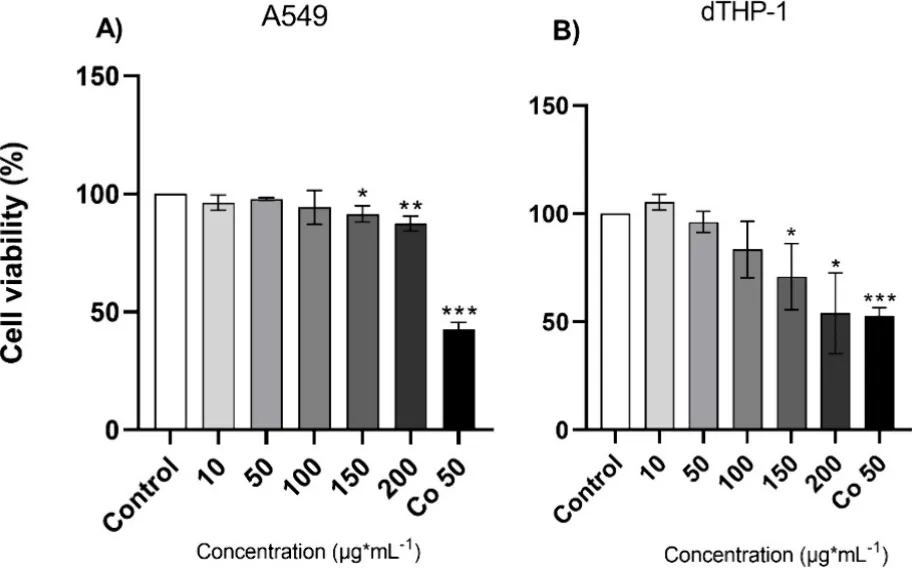
Cell viability of exposed cells under submerged conditions following exposure to PM_2__.5_ from a platform at a subway station in Stockholm. (A) A549 and (B) dTHP-1 exposed (24 h) to 10, 50, 100, 150, and 200 μg/mL of PM_2.5_ subway particles versus controls. Co nanoparticles (Co 50) used as a positive control at 50 μg/mL showed 42 and 52% viability in the A549 and dTHP-1 cells, respectively. Data are presented as mean ± standard deviation of at least three independent exposures. Statistically significant differences are presented as *p ≤ 0.05, **p ≤ 0.01, and ***p ≤ 0.001.

#### 3.2.2 Cytokine Release in ALI

The inflammatory response in A549 monoculture in ALI was not affected, but the LPS (1 μg/mL) positive control also did not increase the release of the measured cytokines (IL-8, IL-6, IL-1β, and TNF-α). Therefore, these results will not be discussed further. In the ALI coculture model, the LPS concentration was increased to 10 μg/mL to ensure a detectable response. While the positive control effectively elicited an inflammatory response, exposure to subway PM_2.5_ emissions did not significantly increase IL-6 or IL-8 levels compared to the Control group (see Figure 5). Due to the high intravariability between exposures, cytokine release data are presented as normalized to control values.

**Figure 5:**
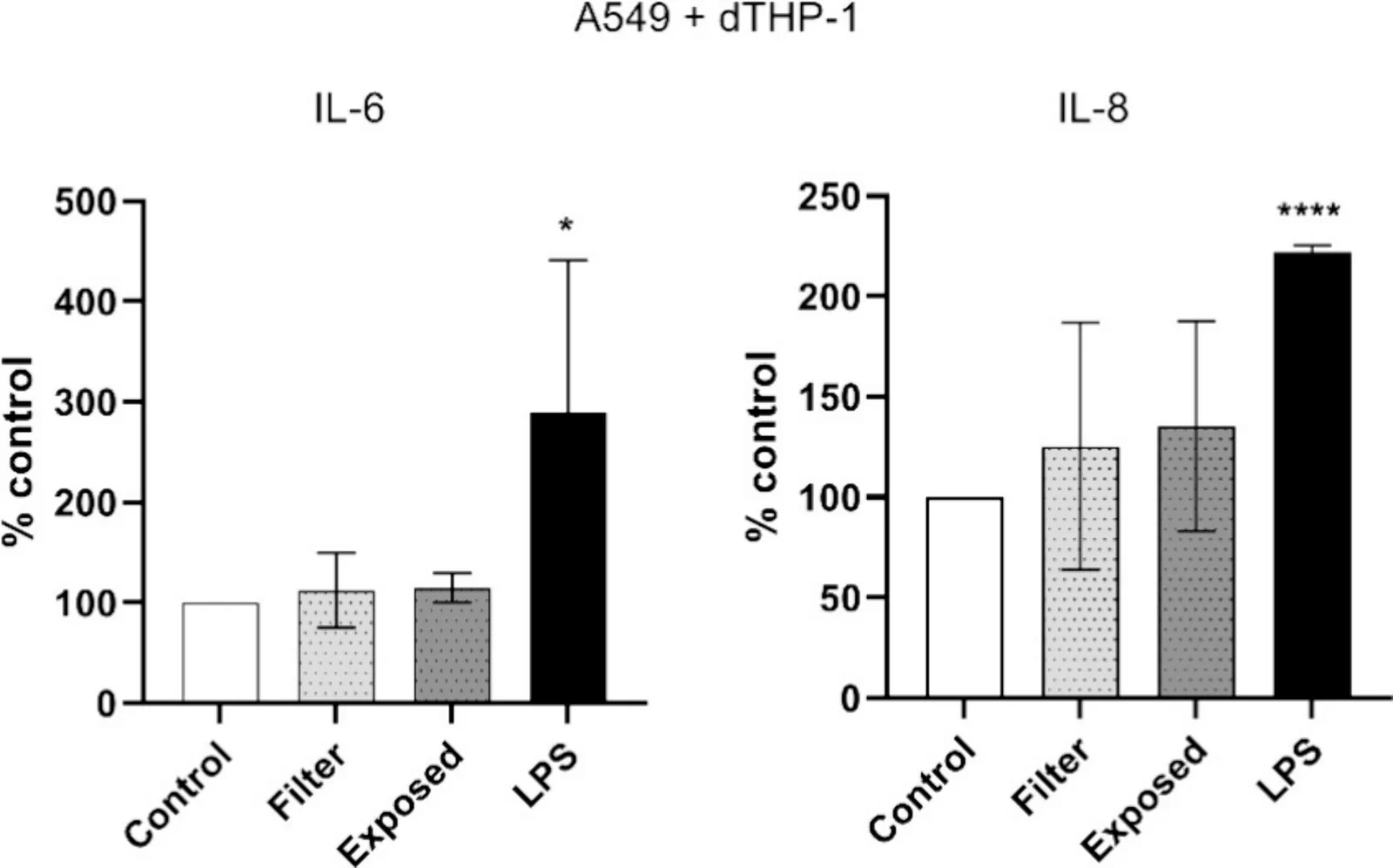
IL-6 and IL-8 release from cocultures (A549 and dTHP-1) in ALI after exposure to subway PM_2.5_ was analyzed with an ELISA assay. After 2 h of exposure and 24 h of incubation, the basal media were used to determine cytokine release. LPS (10 μg/mL) served as a positive control. Data are presented as mean ± standard deviation of at least three independent exposures and expressed as released cytokine versus the control (%). The cell exposure dose of PM_2.5_ at ALI was estimated to be 0.12 ± 0.07 μg/cm^2^. Statistically significant differences are presented as *p* ≤ 0.05. Control = incubator control; Filtered = gas phase only; and Exposed = complete aerosol.

#### 3.2.3 Cytokine Release at Submerged Conditions

Under submerged conditions, exposure to PM_2.5_ from the subway induced a more evident inflammatory response than that observed in the ALI results. After 24 h of incubation with particles, A549 cells exhibited a significant increase of IL-6 and TNF-α at the highest tested dose (100 μg/mL); see Figure 6.

**Figure 6:**
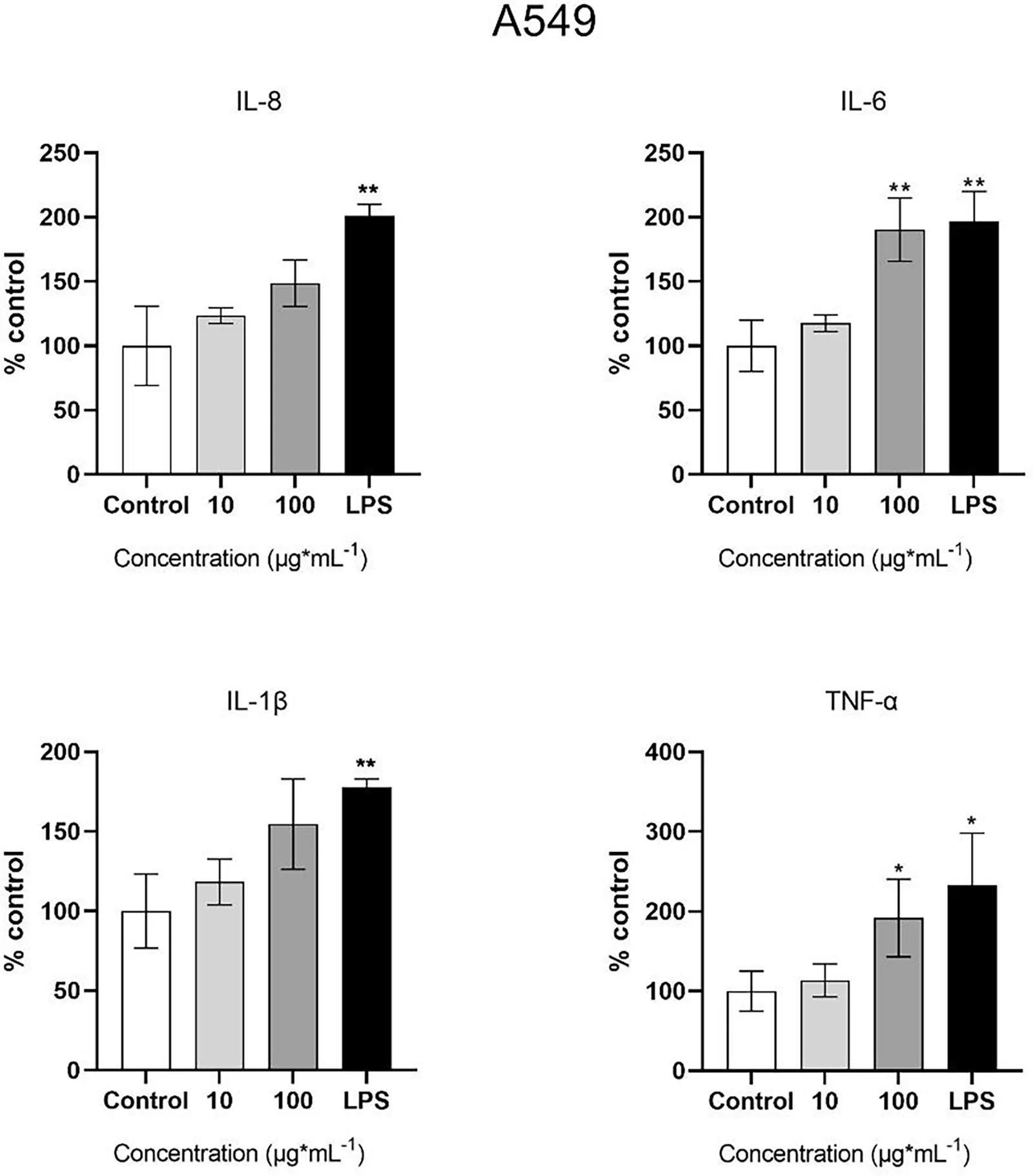
Cytokine release from submerged A549 cells exposed to subway PM_2.5_. Inflammatory cytokines (IL-8, IL-6, IL-1β, and TNF-α) were analyzed using a multiplex cytokine array after 24 h of exposure to 10 and 100 μg/mL of PM_2.5_. LPS (1 μg/mL) served as a positive control. Data are presented as mean ± standard deviation of three independent exposures and expressed as released cytokine versus the control (%). Statistically significant differences are presented as *p ≤ 0.05, **p ≤ 0.01, and ***p ≤ 0.001.

In dTHP-1 cells, IL-8 concentrations were significantly elevated relative to the control even at the lowest exposure dose (10 μg/mL). At the highest dose of subway PM_2.5_ (100 μg/mL), exposure led to a significant increase in all measured cytokines (IL-8, IL-6, IL-1β, and TNF-α); see Figure 7.

**Figure 7:**
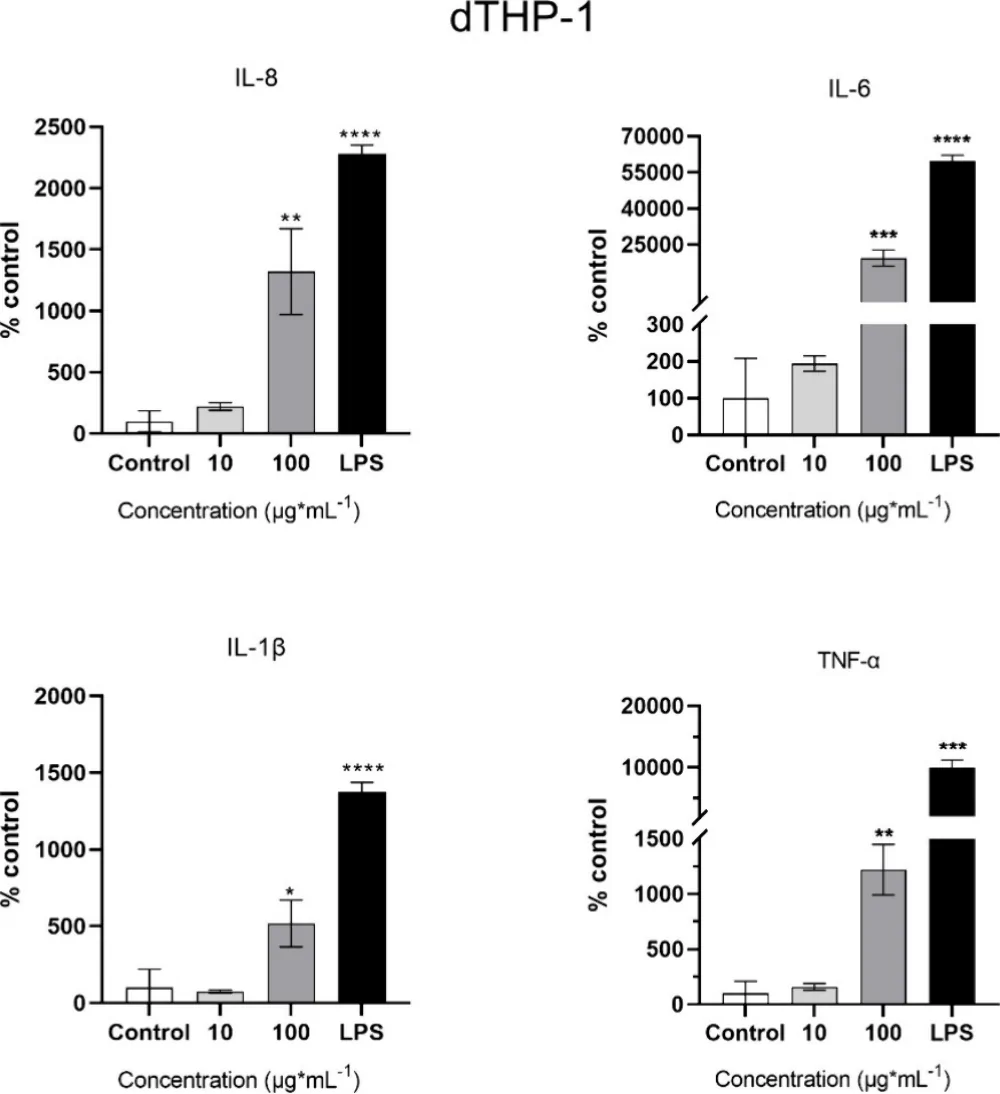
Cytokine release from submerged dTHP-1 cells exposed to subway PM_2.5_. Several cytokines (IL-8, IL-6, IL-1β, and TNF-α) were analyzed using a multiplex cytokine array after 24 h of PM_2.5_ exposure at concentrations of 10 and 100 μg/mL. LPS (1 μg/mL) served as a positive control. Data are presented as mean ± standard deviation of three independent exposures and expressed as released cytokine versus the control (%). Statistically significant differences are presented as *p ≤ 0.05, **p ≤ 0.01, and ***p ≤ 0.001.

### 3.3 Analysis of Inorganic Fraction (SEM–EDS and XRD Analyses)

Iron, O, and C were the most abundant elements in the collected subway PM_2.5_ particles. These elements accounted for more than 90 wt % of the sample. The sample also contained secondary concentrations of Ba, Si, Cu, and S in decreasing amounts (approximately 1–0.5 wt % each); see Table 1.

**Table 1:** Elemental Composition of PM_2.5_ from a Subway Station in Stockholm, Expressed in Weight Percent (wt %)

element	average mass fraction (wt%)	SD
Fe	47.23	1.20
O	25.46	0.90
C	20.52	0.48
Ba	1.15	0.04
Si	1.13	0.04
Cu	0.84	0.04
S	0.57	0.01
Ca	0.49	0.02
Zr	0.48	0.01
Na	0.42	0.02
Cl	0.42	0.03
Mn	0.40	0.01
Al	0.35	0.02
Sn	0.30	0.02
K	0.13	0.02
Mg	0.11	0.00

The crystalline fraction of the PM_2.5_ subway sample was dominated by Fe-based species (Fe, FeO, Fe_2_O_3_, and Fe_3_O_4_), which agrees with the observed elemental composition. In particular, magnetite (α-Fe_3_O_4_) and hematite (α-Fe_2_O_3_) were the most abundant crystalline phases, as can be qualitatively observed by comparing the relative intensities of the corresponding XRD peaks. Additionally, a minor amount of elemental carbon (graphite) and metallic copper was observed; see Figure 8.

**Figure 8:**
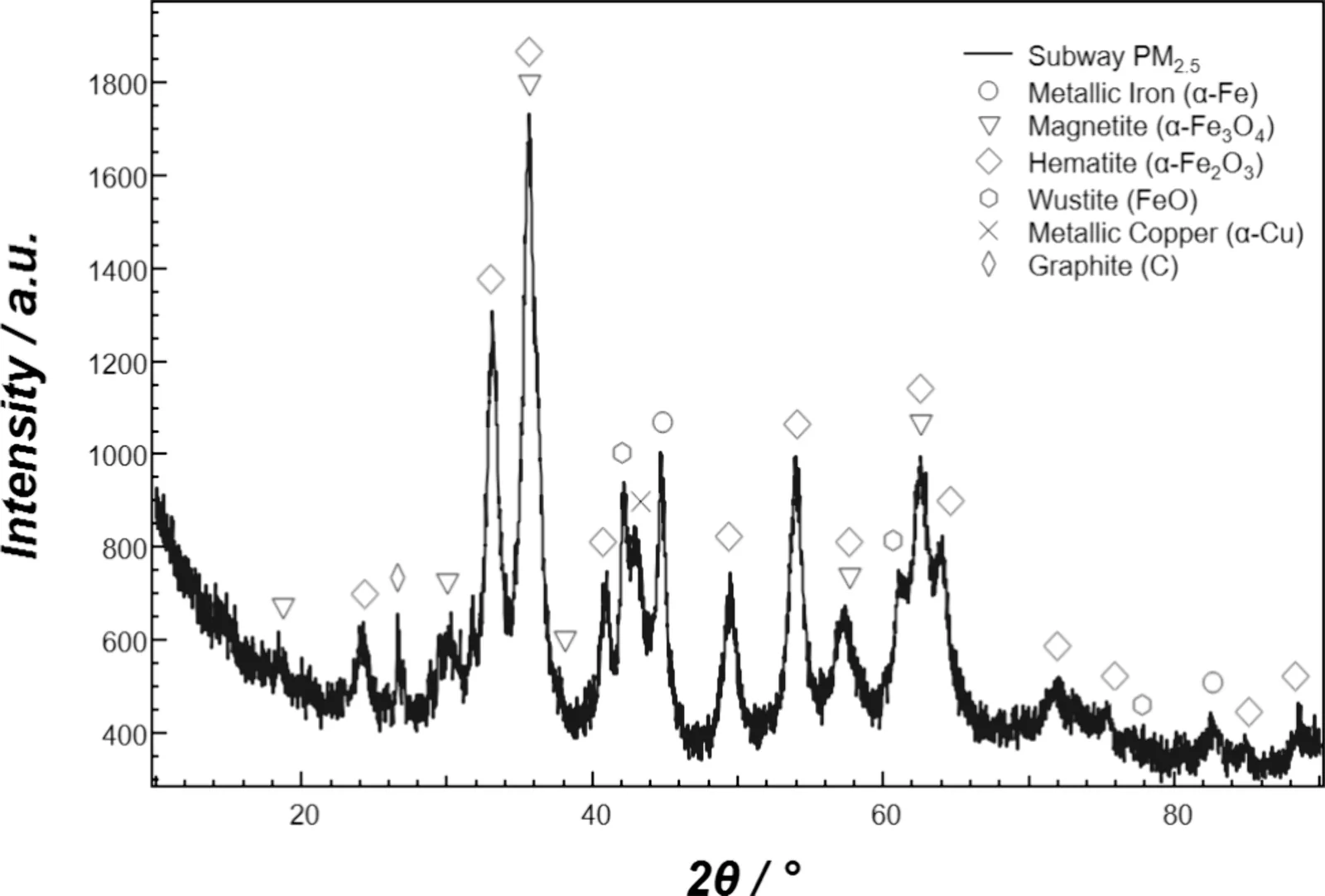
Phase identification of crystalline compounds in subway PM_2.5_ from XRD analysis. Several compounds are observed, including metallic iron, metallic copper, magnetite (α-Fe_3_O_4_), hematite (α-Fe_2_O_3_), wüstite (FeO), and graphite (C).

### 3.4 Analysis of Organic Compounds

Low-molecular-weight PAHs (LMW-PAHs) with 2- and 3-ring structures, medium-molecular-weight PAHs (MMW-PAHs) with 4-ring structures, and high-molecular-weight PAHs (HMW-PAHs) with 5-, 6-, and 7-ring structures, for a total of 24 PAHs, are presented in Table 2. The sum of the quantified PAHs was 48.8 ng/m^3^ based on the air sampled and 896.0 μg/g based on the amount of PM_2.5_ collected.

**Table 2:** Summary of Organic Compound Concentrations Detected in Subway PM_2.5_ ^a^

chemical	abbreviation	concentration (ng/m^3^)	mass fraction (μg/g)
sum of 2- and 3-ring PAHs	LMW-PAHs	8.6	157.1
sum of 4-ring PAHs	MMW-PAHs	28.1	515.1
sum of 5-, 6- and 7-ring PAHs	HMW-PAHs	12.2	223.9
sum of PAHs	∑PAHs	48.8	896.0
sum of OPAHs	∑OPAHs	12.2	0.7
sum of tri-CBs	tri-PCBs	0.05	0.9
sum of tetra-CBs	tetra-PCBs	0.02	0.3
sum of penta-CBs	penta-PCBs	0.04	0.8
sum of hexa-CBs	hexa-PCBs	0.30	5.5
sum of hepta-CBs	hepta-PCBs	0.14	2.6
sum of six indicator PCBs	∑6PCBs	0.21	3.8
sum of PCBs	∑PCBs	0.55	10.1
sum of PBDEs	∑PBDEs	0.06	1.2

a The full list of the chemicals researched is provided in the Supplementary Material, Section S1, Table S1.

The highest concentration was found for MMW-PAHs (28.1 ng/m^3^ and 515.1 μg/g), followed by HMW-PAHs (12.2 ng/m^3^ and 223.9 μg/g) and LMW-PAHs (8.6 ng/m^3^ and 157.1 μg/g). This is probably because LMW-PAHs are generally mainly distributed in the gas phase, while MMW-PAHs and HMW-PAHs are mainly distributed on the particles (Ho et al., 2009). The most abundant PAHs in our sample were fluoranthene, followed by pyrene, benzo­[*e*]­pyrene, and phenanthrene, accounting for 15, 11, and 8% of the total PAHs, respectively, while the other compounds exhibited lower concentrations compared to those PAHs.

∑OPAHs measured in this experiment were 0.7 ng/m^3^ and 12.2 μg/g, with 9-fluorenone and anthraquinone as the most abundant compounds, accounting for almost 65% of the total OPAHs.

Five classes of PCB congeners from tri-CBs to hepta-CBs for a total of 29 congeners including six indicator congeners (PCB 28, 52, 101, 138, 153, and 180) and 11 other toxicologically significant congeners (PCB 77, 81, 105, 114, 118, 123, 156, 157, 167, 169, and 189) were investigated. The concentrations of ∑PCB were 0.55 ng/m^3^ and 10.1 μg/g, normalized to the air sampled and the amount of PM_2.5_ collected, respectively.

Indicators accounted for 38% of the total PCBs. Considering the chlorine distribution, hexa-PCBs accounted for the highest proportion, around 54% of the ∑PCBs, followed by hepta-PCBs, which accounted for 26% of the ∑PCBs. Lower chlorinated PCBs accounted for 3–9% of the ∑PCBs.

Regarding PBDEs, detectable amounts were found for PBDE-47, 99, and 183 and for congeners containing from 8 to 10 bromine atoms. The most abundant congener was BDE-209, and the ∑PBDE concentrations detected were 0.06 pg/m^3^ and 1.2 μg/g based on the volume of air and the amount of PM_2.5_ sampled, respectively.

## 4 Discussion

This study investigated the toxicity of PM_2.5_ particles from the subway system in Stockholm. ALI experiments on the platform exposed A549 and cocultures to complete and filtered aerosols, while PM_2.5_ collected on filters was tested on A549 and dTHP-1 cells under submerged conditions in the laboratory.

In the ALI experiments, A549 and coculture cell viability was not significantly affected by the gas phase (Filtered group) or by exposure to 0.12 ± 0.07 μg/cm^2^ (Exposed group) of subway PM_2.5._ This result was consistent with the observed lack of effect at the lowest concentrations in submerged experiments with A549 and dTHP-1 cell cultures. However, at the higher doses tested in submerged experiments (150–200 μg/mL, which are equal to nominal doses of 46 and 62 μg/cm^2^, respectively), PM_2.5_ from the subway induced a significant decrease in cell viability. The discrepancy in results between ALI and submerged exposures can be attributed to fundamental differences in exposure methodologies, which influence dose delivery, sensitivity across time points for detecting cellular responses, and the contribution of the gas phase present in ALI but absent in submerged conditions.^32^ Furthermore, differences in delivered dose are likely to play an important role. In submerged exposures, cells are typically subjected to higher nominal doses; however, only a fraction of the administered particle concentration ultimately deposits onto and interacts with the cell layer (effective dose). When feasible, the effective dose can be quantified using direct methods, e.g., chemical analysis of the particles. However, the technique is challenging to apply to real-world aerosols, such as subway PM_2.5_, which contain complex mixtures.

Nevertheless, using both methodologies (i.e., ALI and submerged) when testing particle mixtures such as subway PM_2.5_ enables a broader toxicological investigation. Specifically, in this study, submerged exposures enabled controlled dose–response assessments and comparability, while ALI exposures were more physiologically relevant, delivering particles in their airborne state directly to the cell surface, more closely mimicking in vivo inhalation conditions. However, both approaches have limitations, including constraints on achievable doses under field ALI conditions and uncertainties in effective dose estimation in submerged systems.^28,33–35^

In this context, the effective dose in submerged experiments is affected by factors such as particle concentration, morphology, composition, density, agglomeration state, and size, which can significantly impact both the deposition rate and the interaction of the particles with the medium.^36^ Assuming 20% deposition efficiency, as reported by Loxham et al.^18^ for subway particles, the lowest particle suspension concentration of 10 μg/mL in the submerged experiments would yield a cellular dose of 0.62 μg/cm^2^. The average dose in our ALI exposure experiments was five times lower (0.12 ± 0.07 μg/cm^2^). Since both the particle number and mass concentrations in the subway microenvironment remained stable across all ALI exposure experiments, this mean value was considered representative of the PM_2.5_ ALI dose. The lower dose in the ALI exposures, compared to the submerged exposures, likely explains the absence of effects on cell viability. Our findings showing cytotoxicity only at high doses of subway PM_2.5_ align with the results of Seaton et al.^20^ They reported no cytotoxicity (LDH release) in A549 cells after exposure to subway PM_2.5_ at a low dose (10 μg/mL), whereas cytotoxicity increased at higher doses. Similarly, a previous study comparing the cytotoxic effects of subway, subway-intermediate commercial space, and street-level particulate matter observed elevated cytotoxicity in bronchial epithelial cells at high doses (70 μg/mL) of subway particles and even larger effects for outdoor PM fractions.^37^ Interestingly, compared with a previous study conducted under comparable exposure conditions using PM_2.5_ from a road tunnel in Stockholm,^28^ A549 cells in the present study appear to be more sensitive to subway PM_2.5_. Indeed, reduced viability was observed in both A549 and dTHP-1 cells at the two highest tested doses (150 and 200 μg/mL), whereas in the road tunnel study, only dTHP-1 cells were affected. This difference suggests that subway particles can trigger cytotoxic mechanisms affecting cellular targets present in both A549 and dTHP-1 cells (e.g., oxidative stress, DNA damage, and mitochondrial dysfunction).^38,22,24,23^ At the same time, dTHP-1 cells exhibited a greater degree of cell death (at 150–200 μg/mL), suggesting an additional macrophage-specific toxicity, likely related to their phagocytic activity and immune response.^39^

The inflammatory analyses revealed that exposure to gases or PM_2.5_ in ALI did not lead to significant changes in cytokine release in A549 cells. Similarly, in submerged conditions, no alterations in cytokine levels were observed at low doses. However, a notable increase in IL-6 and TNF-α was detected when A549 cells were exposed to 100 μg/mL of particle suspension. These results align with previous findings, where PM fractions did not induce an inflammatory response in primary epithelial cells under either ALI or submerged conditions at doses below 50 μg/mL.^18^ Although A549 and other epithelial cells may exhibit reduced sensitivity to certain toxicological end points compared to bronchial cell lines, e.g., BEAS2-B,^40^ they remain valuable models for alveolar research, as they are a widely used cell line for particulate toxicity.^41,42^ So far, A549 cells have been used to investigate PM from different sources in Stockholm, showing that subway particles induced greater DNA strand breakage, oxidative stress, and DNA damage than street-level particles.^22–24^

Macrophages, such as dTHP-1 cells, are highly suitable models for studying inflammatory responses due to their phagocytic activity, which enhances their interaction with particulate matter.^43^ In this study, we used dTHP-1 cells in both monoculture under submerged conditions and in coculture with A549 cells under ALI conditions (with a ratio of 1:10), as dTHP-1 cells alone do not grow homogeneously in ALI.^44^

No changes in cytokine release were measured in the ALI coculture. In submerged exposures, dTHP-1 monocultures exhibited a strong inflammatory response for all measured cytokines (IL-8, IL-6, IL-1β, and TNF-α) at the highest dose (100 μg/mL), confirming the previously measured pro-inflammatory effect of PM from the subway on macrophages at high doses.^16,17,24^ The absence of cytokine release in the ALI coculture experiments, despite its detection at the lowest dose in submerged dTHP-1 monocultures, can be attributed to both the lower exposure dose in ALI and the reduced number of dTHP-1 cells in the coculture. In this setup, only 10% of the total cell population was dTHP-1, which are the ones most responsive to inflammatory stimuli.

Testing coculture only in ALI represents a limitation, as it prevents a direct comparison between the two exposure models. However, the coculture was implemented only in ALI because it is physiologically relevant at the ALI interface but not under submerged conditions, where cells are fully covered by a medium. Moreover, a previous work has shown that ALI and submerged exposures can differ in sensitivity even when the same cell models are used,^32^ suggesting that the exposure method itself influences cellular responses.

The increased release of TNF-α in A549 cells and, more notably, in dTHP-1 monocultures likely reflects a cellular response to increased oxidative stress caused by the interaction with the particles. Overall, ROS present in particles can stimulate the secretion of pro-inflammatory cytokines, including TNF-α, IL-6, and IL-1β, particularly in immune cells.^45,46^ However, the capacity of subway particles to generate ROS and induce oxidative stress remains contradictory. Several studies have demonstrated that subway particles induce a weaker oxidative stress than street particles,^20,37^ and no clear dose–response relationship in biological markers, for example, in human urine samples, has been observed.^47^ In contrast, other studies have identified a strong correlation between the Fe and other metals in subway particles, with increased oxidative potential and ROS production.^48–50^

Chemical analyses revealed that the samples contained more than 90 mass-% Fe, C, and O, with Fe accounting for approximately 50 mass-%. Traces of Ba, Si, and Cu were detected at concentrations around 1%. These findings align with studies conducted in various American subway stations, the London subway, and earlier studies in the Stockholm subway, where Fe constitutes approximately 50% or more of the total PM_2.5_ composition.^9,23,51,52^ Notably, in Beijing subway station, the Fe content was even higher, and particles contained additional metals such as Al, Mn, Zr, and Cr.^53^ In contrast, the Fe content in Seoul subway particles was lower when sampled at the platform (24.5%), with the particles displaying similar Cu and Mn concentrations to those found at the Stockholm platform. However, particles collected from the Seoul subway, but in the tunnel, exhibited a significantly higher Fe content (43%) and elevated levels of Cu.^54^ These differences suggest that, although the subway system may be considered a relatively isolated environment, the composition of particles is still influenced by various factors, including the sampling location, the specific alloys used in subway components, and the contributions from external sources. Moreover, a recent study investigating only train brake emissions from the Stockholm subway identified a lower Fe concentration in the particle composition (below 30 mass-%) and reported no cytotoxicity or increased cytokine release in A549 and dTHP-1 cells, both in submerged experiments at comparable doses and in ALI exposures at higher doses (4.03 ± 2.48 μg/cm^2^).^55^ Taken together, these observations indicate that train brake particles alone may not fully represent the complexity of subway PM, underscoring the importance of in-field measurements that capture the contributions of multiple sources, including train wheels, rails, as well as the sliding of electric cables.^7^

The metals in particles from different subway systems may also be oxidized to different levels, affecting their reactivity. In Budapest, the Fe in subway PM was predominantly in the form of hematite (α-Fe_2_O_3_) with traces of magnetite (α-Fe_3_O_4_) and exhibited a low toxicity.^56^ In the Korean and Chinese subways, Fe was mainly found in its metallic form and as magnetite.^57,58^ Earlier studies in the Stockholm subway identified magnetite as the predominant iron oxide, which was associated with elevated genotoxicity.^10,23^ Our results indicate that both magnetite and hematite were the dominant oxides in the current Stockholm subway environment. This suggests that emissions from different subway systems, and even within the same system over time, may vary and therefore cause different toxic effects on different *in vitro* models. However, in the present study, it was not possible to corroborate this theory by performing correlation studies between toxicity end points and PM_2.5_ composition, as a single filter was collected and used for both submerged exposures and chemical analyses. Future studies should aim to correlate individual components of PM_2.5_ with toxicological outcomes by extending the duration of ALI exposure and collecting multiple filters.

PAH concentrations in the Stockholm subway were higher compared to those observed in the subway systems of Barcelona and Mexico City^59–61^ and also higher compared to the concentrations measured in a road tunnel in Stockholm.^28^ Since PAHs are primarily emitted from combustion processes,^62^ their concentration in subway environments is not frequently assessed. Nevertheless, their presence is of particular concern, as PAHs are recognized carcinogens that exert pro-tumorigenic effects mainly through activation of the aryl hydrocarbon receptor (AhR) and the release of cytokines from the IL-1 family, thereby promoting inflammatory responses.^63^ In this study, PAHs such as fluoranthene, pyrene, and phenanthrene were detected at concentrations that, in previous research, have been associated with a significant contribution from diesel exhausts to PM_2.5,_ ^64,65^ while benzo­[*e*]­pyrene serves as a marker for atmospheric particle aging.^64–66^ However, during the sampling period in the Stockholm subway, no diesel-powered maintenance trains were operating, and the ventilation system connecting the street level to the tunnel was closed. Under these conditions, the source of the elevated PAH concentrations remains unclear. One speculative nearby source may be a small restaurant located on an intermediate floor between the subway entrance and the platform, as cooking environments have been previously associated with high PAH emissions.^67^

To the best of our knowledge, there is no existing literature on the presence of OPAHs, PCBs, and PBDEs in PM_2.5_ collected from subway environments. However, the OPAH concentrations reported in road tunnels,^68^ as well as in urban and rural areas of China^69^ and several cities in Southern Europe,^65^ are consistent with the concentrations detected in the present study.^70–72^ The abundance of higher chlorinated PCBs in PM_2.5_ suggests a contribution from transformer oil leaks and the disposal of capacitors.^73^ PBDE analysis revealed lower concentrations compared to those found in other public microenvironments, including subway systems.^71^ Among the PBDE congeners, BDE-209 was the most abundant, likely due to the lower light exposure in the subway environment, as BDE-209 is more stable under such conditions. The presence of other BDEs in PM_2.5_ indicates the influence of at least two commercial BDE mixtures.

## 5 Conclusion

In summary, our findings indicate that exposure to PM_2.5_ from subway environments leads to a reduced cell viability and may promote inflammation. The cell viability in submerged experiments decreased in a dose-dependent manner at the highest concentrations. While submerged experiments in A549 showed only partial pro-inflammatory effects, these effects were more evident in dTHP-1 cells, likely due to their immune cell characteristics. Notably, the ALI experiments revealed no significant changes in cell viability or cytokine release in either A549 monocultures or A549 and dTHP-1 cocultures. This lack of response in ALI experiments can be attributed to the lower exposure dose, which was up to five times lower than the lowest dose used in submerged experiments, where no effects were observed in A549. Additionally, the absence of cytokine release in ALI coculture experiments may be attributed to the lower concentration of dTHP-1 cells in the coculture system, which were present at a 1:10 ratio relative to A549 cells. These findings highlight the critical role of exposure conditions in toxicological studies and support the use of both *in situ*- and laboratory-based approaches. Moreover, longer *in situ* exposures may be necessary to achieve doses comparable to those in laboratory setups. Chemical analysis revealed a predominance of iron-based species and high levels of PAHs in subway PM_2.5_ particles.

## Supplementary Material



## Notes

Additional details on the complete analyses of organic compounds measured in PM_2.5_ (Section S1) and the daily variation in PNC in the Stockholm subway (Section S2) (PDF)

## Abbreviations

PM_2.5_: particulate matter smaller than 2.5 μm
ALI: air–liquid interface
A549: human alveolar basal epithelial cell line
dTHP-1: differentiated THP-1 macrophage-like cells
THP-1: human monocytic cell line
IL-6: interleukin 6
IL-8: interleukin 8
IL-1β: interleukin 1 beta
TNF-α: tumor necrosis factor alpha
EPA: Environmental Protection Agency
ROS: reactive oxygen species
ELLIE: electrostatic air–liquid interface exposure system
PNC: particle number concentration
OPC: optical particle counter
CPC: condensation particle counter
PTFE: polytetrafluoroethylene
PBS: phosphate-buffered saline
PMA: phorbol 12-myristate 13-acetate
RPMI-1640: Roswell Park Memorial Institute medium 1640
DMEM: Dulbecco’s modified Eagle's medium
FBS: fetal bovine serum
HEPES: 4-(2-hydroxyethyl)-1-piperazineethanesulfonic acid
SEM–EDS: scanning electron microscopy with energy-dispersive X-ray spectroscopy
XRD: X-ray diffraction
GPC: gel permeation chromatography
SPE: solid phase extraction
PAHs: polycyclic aromatic hydrocarbons
OPAHs: oxygenated polycyclic aromatic hydrocarbons
PCBs: polychlorinated biphenyls
PBDEs: polybrominated diphenyl ethers
LMW-PAHs: low-molecular-weight polycyclic aromatic hydrocarbons
MMW-PAHs: medium-molecular-weight polycyclic aromatic hydrocarbons
HMW-PAHs: high-molecular-weight polycyclic aromatic hydrocarbons
AhR: aryl hydrocarbon receptor
SD: standard deviation
LPS: lipopolysaccharide
nPETS: Nanoparticle Emissions from the Transport Sector (EU Horizon 2020 project)
