# Supplementary material for: Novel Approach to Assess the Toxicity of PM2.5 on a Subway Platform Using a Mobile Air–Liquid Interface System and Submerged Exposure
Source: ACS EST Air. 2026 Jul 1;3(8):1976–87. doi: 10.1021/acsestair.6c00018 (PMC13491529; doi:10.1021/acsestair.6c00018)
Supplement: Supplementary file 1 [file ea6c00018_si_001.pdf]

Supporting Information for Publication

Novel approach to assess the toxicity of $PM_{2.5}$ on a subway platform using a mobile Air Liquid Interface system and submerged exposure

Micol Introna[1*], Ana Teresa Juárez-Facio[1], Naga Veera Srikanth Vallabani[2], Minghui Tu[3], Michael Norman[4], Sanna Silvergren[4], Andrea Colombo[5], Valentina Liboni[5], Bozhena Tsyupa[6], Alessandro Mancini[6], Ulf Olofsson[3], Sarah Sulamith Steimer[1], Hanna Lovisa Karlsson[2], Karine Elihn[1]

[1]Department of Environmental Science, Stockholm University, Stockholm, 106 91, Sweden.
[2]Institute of Environmental Medicine, Karolinska Institute, Stockholm, 171 77, Sweden
[3]Department of Machine Design, KTH Royal Institute of Technology, Stockholm, 100 44, Sweden
[4]Environment and Health Administration, SLB-analys, Stockholm, 104 20, Sweden
[5]Department of Environmental Health Sciences, Istituto di Ricerche Farmacologiche "Mario Negri"- IRCCS, Milano, 20156, Italy
[6]Materials Engineering & Laboratories, GCF R&D, Brembo NV, Stezzano (BG), 24040, Italy
*Corresponding author: micol.introna@aces.su.se

Section S1: *Complete analysis of organic compounds*

The concentrations of the organic compounds measured in $PM_{2.5}$ are presented in Table S1 (concentrations expressed in µg/g and in ng/m³).

Table S1: *Complete list of all analyzed organic compounds in $PM_{2.5}$ collected on the subway platform in Stockholm, expressed in µg/g and in ng/m³.*

City	Stockholm
Sampling Site	Subway
Start	19-12-22
End	23-12-22

**Compound**	**Abbreviation**	**Concentration (µg/g)**	**Concentration (ng/m³)**
Naphtalene	Np	17.5	0.95
Acenaphthylene	Acy	1.9	0.10
Acenaphtene	Ace	1.3	0.07
Fluorene	F	3.3	0.18
Phenantrene	Ph	69.5	3.79
Anthracene	An	5.1	0.28
1-mPhenanthrene	1-MEPh	19.5	1.07
2-mPhenanthrene	3-MEPh	26.3	1.43
3-mPhenanthrene	2-MEPh	7.1	0.39
9-mPhenanthrene	9-MEPh	5.6	0.30
Fluoranthene	Fl	315.7	17.21
Pyrene	Py	138.3	7.53
Benz[a]anthracene	B[a]An	17.3	0.94
Triphenylene	TP	11.1	0.60
Chrysene	Chr	32.8	1.79
Benzo[b]fluoranthene	B[b]Fl	29.8	1.63
Benzo[k]fluoranthene	B[k]Fl	14.9	0.81
Benzo[j]fluoranthene	B[j]Fl	14.4	0.79
Benzo[e]pyrene	B[e]Py	97.0	5.29
Benzo[a]pyrene	B[a]Py	22.7	1.24
Indeno[1,2,3-cd]pyrene	I[1,2,3-cd]Py	16.8	0.92
Benzo[ghi]perylene	B[ghi]Pe	21.3	1.16
Dibenz[a,h]anthracene	dB[a,h]An	3.8	0.21
Coronene	COR	3.0	0.16
1,4-naphtoquinone	(1,4)O2NAP	0.88	0.05
9-fluorenone	9-OFLN	3.43	0.19
Acenaphtoquinone	AceNQ	< 0.02	< 0.01
Antraquinone	(9,10)O2ANT	4.44	0.24

2-nitrofluorenone	2-N-9-OFLN	< 0.02	< 0.01
Benzo(a)fluorenone	BaOFLN	1.58	0.09
Benzo(a)anthrone	BAN	0.96	0.05
1,2 benzoantraquinone	O2BAA	0.85	0.05
3,4,4',5-Tetrachlorobiphenyl	PCB 81	< 0.001	< 0.01
3,3',4,4'-Tetrachlorobiphenyl	PCB 77	< 0.006	< 0.01
2',3,4,4',5-Pentachlorobiphenyl	PCB 123	< 0.002	< 0.01
2,3',4,4',5-Pentachlorobiphenyl	PCB 118	0.179	0.01
2,3,4,4',5-Pentachlorobiphenyl	PCB 114	< 0.003	< 0.01
2,3,3',4,4'-Pentachlorobiphenyl	PCB 105	0.044	< 0.01
3,3',4,4',5-Pentachlorobiphenyl	PCB 126	< 0.001	< 0.01
2,3',4,4',5,5'-Hexachlorobiphenyl	PCB 167	0.051	< 0.01
2,3,3',4,4',5-Hexachlorobiphenyl	PCB 156	0.119	0.01
2,3,3',4,4',5'-Hexachlorobiphenyl	PCB 157	0.012	< 0.01
3,3',4,4',5,5'-Hexachlorobiphenyl	PCB 169	< 0.001	< 0.01
2,3,3',4,4',5,5'-Heptachlorobiphenyl	PCB 189	0.013	< 0.01
2,4,4'-Trichlorobiphenyl	PCB 28	0.890	0.05
,2',5,5'-Tetrachlorobiphenyl	PCB 52	0.299	0.02
2,2',3,5',6-Pentachlorobiphenyl	PCB 95	0.174	0.01
2,2',4,5,5'-Pentachlorobiphenyl	PCB 101	0.202	0.01
2,2',4,4',5-Pentachlorobiphenyl	PCB 99	0.041	< 0.01
2,3,3',4',6-Pentachlorobiphenyl	PCB 110	0.152	0.01
2,2',3,5,5',6-Hexachlorobiphenyl	PCB-151	0.875	0.05
2,2',3,4',5,5'-Hexachlorobiphenyl	PCB-146	< 0.001	< 0.01
2,2',3,4',5',6-Hexachlorobiphenyl	PCB 149	2.779	0.15
2,2',4,4',5,5'-Hexachlorobiphenyl	PCB 153	0.842	0.05
2,2',3,4,4',5'-Hexachlorobiphenyl	PCB 138	0.713	0.04
2,2',3,3',4,4'-Hexachlorobiphenyl	PCB 128	0.066	< 0.01
2,2',3,4,4',5',6-Heptachlorobiphenyl	PCB-183	0.001	< 0.01
2,2',3,4',5,5',6-Heptachlorobiphenyl	PCB-187	0.339	0.02
2,2',3,3',4',5,6-Heptachlorobiphenyl	PCB 177	0.800	0.04
2,2',3,4,4',5,5'-Heptachlorobiphenyl	PCB-180	0.879	0.05
2,2',3,3',4,4',5-Heptachlorobiphenyl	PCB 170	0.584	0.03
2,4-Dibromodiphenyl ether	BDE-7	< 0.001	< 0.01
4,4'-Dibromodiphenyl ether	BDE-15	< 0.001	< 0.01
2,2',4'-Tribromodiphenyl ether	BDE-17	< 0.001	< 0.01
2,4,4'-Tribromodiphenyl ether	BDE-28	< 0.001	< 0.01

2,2',4,5'-Tetrabromodiphenyl ether	BDE-49	< 0.001	< 0.01
2,3',4',6-Tetrabromodiphenyl ether	BDE-71	< 0.001	< 0.01
2,2',4,4'-Tetrabromodiphenyl ether	BDE-47	0.080	< 0.01
2,3',4,4'-Tetrabromodiphenyl ether	BDE-66	< 0.001	< 0.01
3,3',4,4'-Tetrabromodiphenyl ether	BDE-77	< 0.001	< 0.01
2,2',4,4',6-Pentabromodiphenyl ether	BDE-100	< 0.001	< 0.01
2,3',4,4',6-Pentabromodiphenyl ether	BDE-119	< 0.001	< 0.01
2,2',4,4',5-Pentabromodiphenyl ether	BDE-99	0.029	< 0.01
2,2',3,4,4'-Pentabromodiphenyl ether	BDE-85	< 0.001	< 0.01
3,3',4,4',5-Pentabromodiphenyl ether	BDE-126	< 0.001	< 0.01
2,2',4,4',5,6'-Hexabromodiphenyl ether	BDE154	< 0.001	< 0.01
2,2',4,4',5,5'-Hexabromodiphenyl ether	BDE-153	< 0.001	< 0.01
2,2',3,4,4',5'-Hexabromodiphenyl ether	BDE-138	< 0.001	< 0.01
2,3,3',4,4',5-Hexabromodiphenyl ether	BDE-156	< 0.001	< 0.01
2,2',3,4,4',6,6'-Heptabromodiphenyl ether	BDE-184	< 0.001	< 0.01
2,2',3,4,4',5',6-Heptabromodiphenyl ether	BDE-183	0.007	< 0.01
2,3,3',4,4',5',6-Heptabromodiphenyl ether	BDE-191	< 0.001	< 0.01
2,2',3,3',4,4',6,6'-Octabromodiphenyl ether	BDE-197	0.004	< 0.01
2,2',3,3',4,4',5,6'-Octabromodiphenyl ether	BDE-196	0.006	< 0.01
2,2',3,3',4,4',5,6,6'-Nonabromodiphenyl ether	BDE-207	0.018	< 0.01
2,2',3,3',4,4',5,5',6-Nonabromodiphenyl ether	BDE-206	0.033	< 0.01
Decabromodiphenyl ether	BDE-209	0.984	0.05

$PM_{2.5}$ filters were placed in 33 mL stainless steel extraction cells and fortified with a mixture of internal standards. These included 17 deuterated PAHs, 2 deuterated OPAHs (Augsburg, Germany), 20 13C12-labeled PCBs, and 12 13C12-labeled PBDEs (Wellington Laboratories, Ontario, Canada). Diatomaceous earth was used to fill the remaining cell volume. Extraction was performed using an ASE 350 system (Dionex, Sunnyvale, CA, USA). The first extraction employed dichloromethane, followed by a second with a 1:1 acetone–dichloromethane mixture to target polar compounds. Conditions included a 5 min preheat, three 15 min static cycles at 1500 psi, 60% flush volume, and a 100 s nitrogen purge. Combined extracts were concentrated for subsequent clean-up.

The extract was reduced to 5 mL and injected into a 5 mL loop of an automated GPC system (AccuPrep MPS™, J2 Scientific). Separation was achieved using a 2.5 × 43 cm column packed with 40 g BioBeads SX-3 resin, operated at 5 mL/min with dichloromethane. Calibration followed EPA Method 3640. The eluate (50 mL) was collected between 15:30 and 25:30 min and concentrated to 5 mL.

A 1 mL aliquot (20%) was concentrated to 30 µL and spiked with 20 µL benzo[a]pyrene-D12 for PAH/OPAH analysis. The remaining 4 mL (80%) was reduced to 0.5 mL, diluted to 4 mL with hexane, and subjected to automated clean-up (Dioxin/SPE module) using acid/neutral silica and basic alumina cartridges. After discarding the initial 75 mL n-hexane fraction, the target fraction was eluted with 120 mL of 1:1 n-hexane–dichloromethane, evaporated to near dryness, and reconstituted with 20 µL of 13C12-labeled PCB and PBDE recovery standards.

Quantification was performed using a Thermo Scientific Orbitrap Exploris GC 60K system equipped with a programmable temperature vaporizing (PTV) injector and TriPlus RSH autosampler. Chromatographic separation was achieved using a TRACE 1310 GC with a BPX-DXN column for PAHs, OPAHs, and PCBs, and an Rtx-1614 column for PBDEs.

For PAHs, OPAHs, and PCBs, 2 µL of sample was injected at 75°C, ramped to 320°C at 300°C/min. The oven program was: 100°C (1 min), ramp to 270°C at 10°C/min, then to 320°C at 3°C/min, held for 10 min. For PCBs: 125°C (2 min), ramp to 190°C at 7.5°C/min, then to 280°C at 2.5°C/min, held for 7 min. For PBDEs, 2 µL was injected in splitless mode at 290°C; the oven was programmed from 100°C (5 min), ramped to 325°C at 10°C/min, held for 20 min. The MS operated in electron impact mode (70 eV for PAHs/PCBs, 30 eV for PBDEs) and in Selected Ion Monitoring (SIM) mode.

Compound identification was based on retention times, mass spectra, and comparison with authentic standards and spectral libraries. Halogenated compound peaks were accepted if the isotopic ratio deviated by no more than ±15% from the internal standard. Theoretical isotopic ratios and acceptance ranges are reported in Tables S6 and S7.

Limits of detection (LODs) and quantification (LOQs) were determined per sample using signal-to-noise ratios of 3:1 and 10:1, respectively. Procedural blanks were analyzed to assess background contamination. Quantification was based on five-point calibration curves (1–1000 pg/µL for

PAHs/OPAHs; 0.1–200 pg/μL and 1–500 pg/μL for PCBs and PBDEs, respectively), with correlation coefficients ranging from 0.970 to 0.999.

Data acquisition and processing were performed using Thermo Scientific Chromeleon software, which integrates instrument control, method development, and quantitative analysis.

Section S2: *Daily particle number concentration on the subway platform.*

The daily variation in Particle Number Concentration (PNC expressed as #/cm^3) in the Stockholm Subway from March to June 2023 is presented in Figure S1.

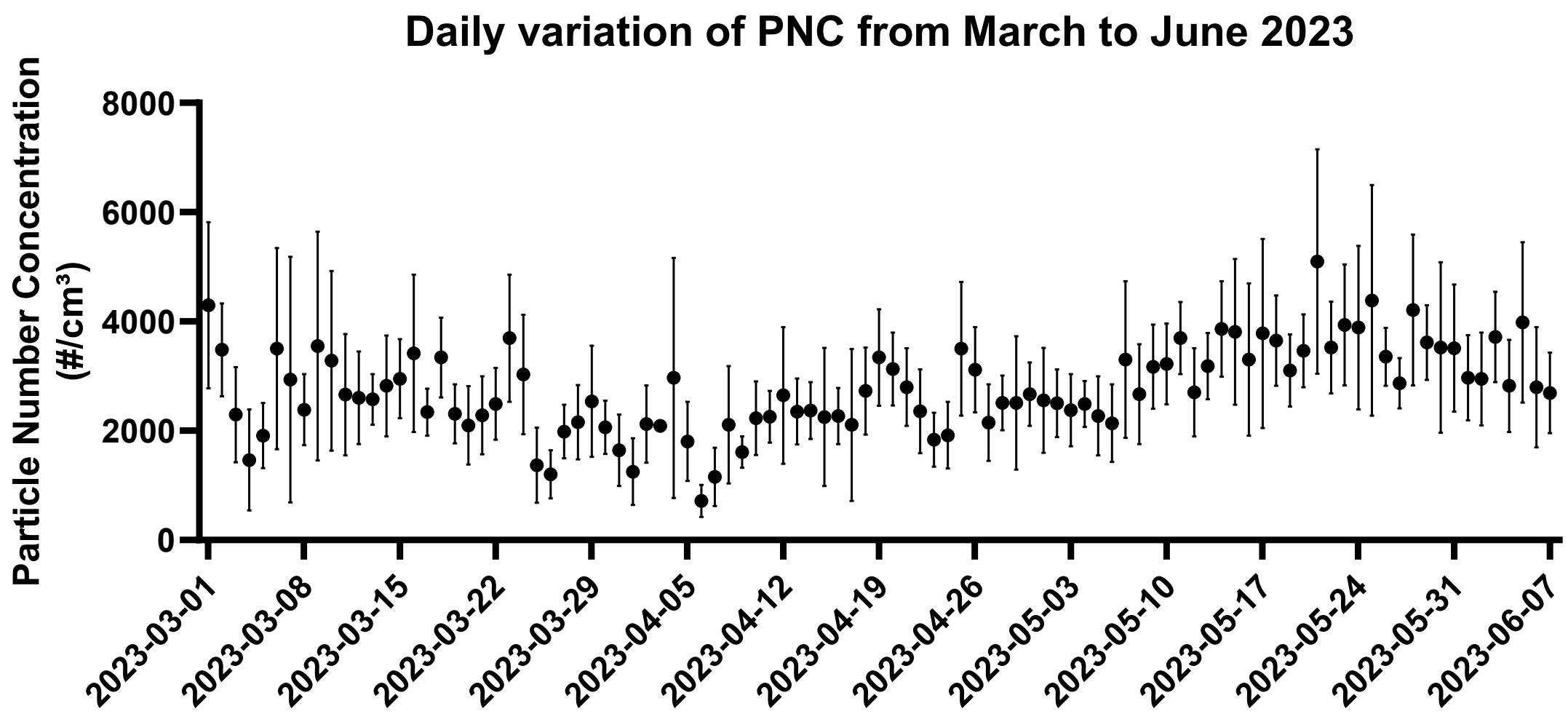

Figure S1. *Daily variation of Particle Number Concentration (PNC, expressed as #/cm³) in the Stockholm subway from March to June 2023 from 9 AM to 12 PM. Values represent the mean ± standard deviation (SD). The x-axis indicates the experiment dates.*
